# Performance of Feature Selection Methods

**DOI:** 10.2174/138920209789177629

**Published:** 2009-09

**Authors:** Edward R Dougherty, Jianping Hua, Chao Sima

**Affiliations:** 1 Department of Electrical and Computer Engineering, Texas A&M University, College Station, TX, USA; 2 Computational Biology Division, Translational Genomics Research Institute, Phoenix, AZ, USA; 3 Department of Pathology, University of Texas M. D. Anderson Cancer Center, Houston, TX, USA

## Abstract

High-throughput biological technologies offer the promise of finding feature sets to serve as biomarkers for medical applications; however, the sheer number of potential features (genes, proteins, etc.) means that there needs to be massive feature selection, far greater than that envisioned in the classical literature. This paper considers performance analysis for feature-selection algorithms from two fundamental perspectives: How does the classification accuracy achieved with a selected feature set compare to the accuracy when the best feature set is used and what is the optimal number of features that should be used? The criteria manifest themselves in several issues that need to be considered when examining the efficacy of a feature-selection algorithm: (1) the correlation between the classifier errors for the selected feature set and the theoretically best feature set; (2) the regressions of the aforementioned errors upon one another; (3) the peaking phenomenon, that is, the effect of sample size on feature selection; and (4) the analysis of feature selection in the framework of high-dimensional models corresponding to high-throughput data.

## INTRODUCTION

High-throughput technologies for genomics and proteomics offer the ability to simultaneously measure vast numbers of biological variables, thereby providing enormous amounts of multivariate data with which to discriminate between phenotypes, such as those corresponding to different variants of a disease, different stages of a disease, different survival rates, and different responses to a drug. While at first glance it seems beneficial to have measurements for the expression levels of thousands of genes or proteins, the benefit can only be realized if there is a sufficiently large sample to avoid overfitting the data, which means that a classifier designed from the data can satisfactorily discriminate between the classes in the data but that this discrimination does not extend to the general populations from with the classes have been chosen, thereby making the classifier useless for new data points, exactly the data points in which we are truly interested. Overfitting is exacerbated by designing a classifier that is too complex for the amount of available data. One cause of excessive complexity is too many features. Thus, in high-dimensional classification problems, when sample size is limited, it is commonplace to constrain the number of features.

Constraining the number of features results in a subfamily of the original family of classifiers. For instance, if there are *d* features available, then the designed classifier is chosen from among all functions of the form ψ(*x_1_*, *x_2_*,…, *x_d_*), where the form of ψ depends on the rule chosen for classifier design. If feature constraint is employed so that the final designed classifier is limited to *k* features, *k* < *d*, then the designed classifier is chosen from among all functions of the form ψ(*x_i_*_1_, *x_i_*_2_,…, *x_ik_*), where $\left\{x_{i1,}x_{i2,...,}x_{\mathrm{ik}}\right\}\subset \left\{x_{1,}x_{2,...,}x_{d}\right\}$ . The form of the classifier remains but with feature constraint the final classifier must operate on a *k*-dimensional subspace of the original *d*-dimensional space. This reduction in the size of the function space from which a classifier is chosen reduces the likelihood of overfitting.

Numerous feature-selection algorithms have been proposed during the last few decades. In the era of high-throughput genomic technology, many more have been added to the repository [1, 2]. Compared to the explosion in proposed methods, there has been little research on the properties of feature-selection algorithms and insufficient attention paid to rigorous performance analysis. Only a few extensive comparative studies have been conducted. A brief summary of the data and validation schemes in four of these are listed in Table **1**. (Only studies on two-class classification are included; studies on multi-class classification problems, like [3], and on feature extraction, like [4], are not discussed). The early studies [5, 6] preceded the emergence of high-throughput genomic studies and emphasize more general problems. Except Kittler’s synthetic data, which are distributed as a 20-dimension Gaussian distribution, all other data sets for comparison are from real data: for example, in [6], the SAR dataset contains 10 texture features that represent 3 different landmarks and the vehicle dataset contains 10 features extracted from silhouette images of 4 different types of cars. Most features are well-selected or extracted from raw data and their number is limited, well below 100 in all cases. Moreover, owing to easy access of the data, there are usually plenty of sample points; hundreds or even thousands are common. The two recent papers [7, 8] are microarray-based studies and all datasets are from real data where the feature size is usually several thousands or more, and sample size is quite limited, less than 200 in all data sets. Except for one real dataset, all four studies applied cross-validation-based approaches on available datasets, as can be seen from the Criterion Function column. Using cross-validation to validate feature-selection algorithms is risky owing to the high variance of cross-validation [9], which is exacerbated in the presence of feature selection [10, 11].

Here we consider performance criteria for feature-selection algorithms arising from two fundamental perspectives: (1) How does the classification accuracy achieved with a selected feature set compare to the accuracy when the best feature set is used? (2) What is the optimal number of features that should be used and to what extent is performance impacted if this optimal number is not used? Inherent in the latter criterion is the analysis of peaking in feature selection, which refers to the tendency of obtaining improved classification accuracy with an increasing number of features only to a point, after which more features result in poorer classification. Our interest is in performance criteria and we refer to the cited literature for more extensive application of these criteria.

## BACKGROUND

Classification involves a *feature vector* **X** = (*X*_1_, *X*_2_,..., *X_d_*) on *d*-dimensional Euclidean space ${\text{ℜ}}^{d}$ composed of random variables (*features*), a binary random variable *Y*, and a function (*classifier*) ψ: ${\text{ℜ}}^{d}$ → {0, 1} to predict *Y*, which means that *Y* is to be predicted by ψ(**X**). The values, 0 or 1, of *Y* are treated as class *labels*. *X*_1_, *X*_2_,..., *X_d_* can be discrete or real-valued. A classifier partitions the feature space ${\text{ℜ}}^{d}$ into two classes, ℜ0=x:ψx=0and ℜ1=x:ψx=1 .Equivalently, a classifier is defined by such a partition. The error, ε[ψ], of ψ is the probability that the classification is erroneous, namely, ε[ψ] = *P*(ψ(**X**) ≠ *Y*). Classifier error depends on the probability distribution, *F*_**x**,*y*_(**x**, *y*), called the *feature-label distribution*, of the feature-label pair (**X**, *Y*). Classification accuracy depends on how well the *class conditional distributions*, *F*_**x**|0_(**x**) and *F*_**x**|1_(**x**), are separated by the partition $\left\{{ℜ}_{0},{ℜ}_{1}\right\}$ . If *A* is the class of all binary functions on ${ℜ}_{d}$ , then an optimal classifier ψ_A_ is one minimizing $\left\{\varepsilon \left[\text{ψ}\right]:\text{ψ}\in A\right\}\text{,}$ and $\varepsilon \left[\psi_{A}\right]=\text{min}\left\{\varepsilon \left[\psi \right]:\psi \in A\right\}$ . $\psi_{A}$ and $\varepsilon_{A}$ are called the *Bayes classifier* and *Bayes error*, respectively.

In practice, the feature-label distribution is unknown and a classifier ψ_*n*_ is designed from a random sample *S_n_* = {(**X**_1_, *Y*_1_), (**X**_2_, *Y*_2_),…, (**X**_*n*_, *Y_n_*)} of vector-label pairs drawn from a feature-label distribution by a classification rule operating on random samples. A *classification rule* is a mapping $\Psi :{\left[{ℜ}^{d}\times \left\{0,1\right\}\right]}^{n}\to A$ Given a sample *S_n_*, we obtain a designed classifier ψ_*n*_ = Ψ(*S_n_*). Unless ψ_*n*_ happens to be a Bayes classifier, its error, ε_*n*_, exceeds the Bayes error so that there is a *design cost* $\Delta_{n}=\varepsilon_{n}-\varepsilon_{A}$ , where ε_*n*_ and Δ_*n*_ are sample-dependent random variables. The expected design cost is *E*[Δ_*n*_], the expectation being relative to all possible samples. The expected error of ψ_*n*_ is decomposed as $E\left[\varepsilon_{n}\right]=\varepsilon_{A}+E\left[\Delta_{n}\right]$ . If *E*[Δ_*n*_] → 0 as *n* → ∞, then the rule is said to be *consistent* relative to the feature-label distribution. If *E*[Δ_*n*_] → 0 as *n* → ∞ for any feature-label distribution, then the rule is said to be *universally consistent*.

A basic problem of classifier design is that the expected design cost is too high when samples are small. Intuitively, this occurs because classification rules often are constructed with the idea in mind that the sample data represent the feature-label distribution. Since small samples tend to provide poor representation of the full distribution, a designed classifier may perform well on the data but poorly on the feature-label distribution. In other words, upon designing a classifier on a particular data set, {(**x**_1_, *y*_1_), (**x**_2_, *y*),…, (**x**_*n*_, *y_n_*)}, the error, ε_*n*_[ψ_*n*_] =  |{ψ_*n*_(**x**_*j*_) ≠ *y_j_*: *j* = 1, 2,…, *n*}|/*n*, of the classifier on the data set, called the *resubstitution* error, may be small but the true error of the classifier may be large. Overfitting of the sample data can result in large expected design cost. As the sample size grows, the sample data tend to better represent the feature-label distribution.

Constraining the classifier to a smaller class, *C*, of potential classifiers can reduce the expected design cost. Limiting the family of classifiers limits the possible partitions, thereby limiting the degree to which a partition can conform to the data, and thus mitigating overfitting. The design cost is now relative to the optimal constrained classifier, Δ_*C,n*_ = ε_*n*_ - ε_*C*_, but now there is a constraint cost, $\Delta_{c}=\varepsilon_{c}-\varepsilon_{A}$ . We can write the expected error of the designed classifier as

(1) $$E\left[{\text{ε}}_{n}\right]={\text{ε}}_{A}+{\text{Δ}}_{c}+E\left[{\text{Δ}}_{c,n}\right]$$

A constraint is beneficial if the reduction in expected design cost more than offsets the constraint cost. With constraint, consistency is relative to both the distribution and the constraint.

With feature constraint, classifiers are constrained to operate on *k*-dimensional subspaces of ${ℜ}^{d}$ .There is a natural ordering of constraints. If C_*k*_ denotes the set of all classifiers on *k*-dimensional subspaces, then *C*_1_ ⊂ *C*_2_ ⊂ … ⊂ *C*_*d*_. There is a corresponding ordering of the errors of optimal classifiers with 1, 2,…, *d* features, namely, ε_*c_1_*_ ≥ ε_*c_2_*_ ≥...≥ ε_*C_d_*_. But this ordering does not extend to designed classifiers on account of design cost. Equation 1 takes the form

(2) $$E\left[{\text{ε}}_{n,k}\right]={\text{ε}}_{A}+{\text{Δ}}_{c_{k}}+E\left[{\text{Δ}}_{c_{k},n}\right]$$

Δ_*c_1_*_ ≥ Δ_*c_2_*_ ≥ ... ≥ Δ_*c_d_*_ and, assuming that the expected design cost increases with more features, *E*[Δ_*c_1_,n*_] ≤ *E*[Δ_*c_2_,n*_] ≤ ... ≤ *E*[Δ_*c_d ,n_*_]. The behavior of *E*[ε_*n*,*k*_], which determines the benefit of feature constraint, depends on the sizes of the constraint and expected design costs, the rate of decrease for constraint cost, and rate of increase for expected design cost.

Often, the expected error of the designed classifier first decreases and then increases with an increasing number of features. This convex behavior is called the *peaking phenomenon* because the benefit of feature constraint peaks (lowest expected error) at some optimal number of features and then increases thereafter [12-17]. In general, the behavior of the expected error relative to the number of features is more complicated and depends on the classification rule and feature-label distribution. In addition, it is often thought that the optimal number of features increases as the sample size increases, but again the general situation is more complicated.

Given a sample, in principle one could consider all feature sets of sizes 1 through *d*, design the classifier corresponding to each feature set, and choose the feature set, *A_n_*_,*k*_, whose designed classifier, ψ_*n*,*k*_, has the smallest error, ε_*n*,*k*_. The first problem with this exhaustive search is computational: too many classifiers to design. Moreover, a full search cannot be avoided if we want to assure optimality, because to select a subset of *k* features from a set of *d* features and be assured that it provides an optimal classifier with minimum error among all classifiers for feature sets of size *k*, all *k*-element subsets must be checked unless there is distributional knowledge that mitigates the search requirement [18]. Second, since the errors of all designed classifiers over all feature sets have to be estimated, inaccurate estimation can lead to poor feature-set ranking – a problem exacerbated with small samples. To address the computational limitations numerous suboptimal feature-selection algorithms have been proposed. Some are affected by error estimation; others are impacted by the need to estimate parameters used in the selection process.

## BEST FEATURE SETS

A direct way of analyzing feature-selection performance involves comparing the performances of a selected feature set, *A_n_*_,*k*_, and a sample-independent best feature set, $A_{k}^{\mathrm{best}}$ by comparing the error, ε_*n*,*k*_, of the classifier, ψ_*n*,*k*_, designed for *A_n_*_,*k*_ by the classification rule, including feature selection, with the error, ${\text{ε}}_{n,k}^{\mathrm{best}}$ , of the classifier, ${\text{ψ}}_{n,k}^{\mathrm{best}}$ , designed for $A_{k}^{\mathrm{best}}$ by the classification rule, absent feature selection. Since, in practice, all that is available is the sample data, it is natural to design the classifiers on the sample data. Since feature selection is the issue, their true errors are computed using the feature-label distribution. The errors are random variables relative to the random sampling. Their relationship is characterized by the joint distribution of the random vector $\left({\text{ε}}_{n,k,}{\text{ε}}_{n,k}^{\mathrm{best}}\right)$ .If one is only interested in the features, not how they function relative to sample data, then an alternative is to design the classifiers on the feature-label distribution itself.

How do we find the best feature set? First, suppose no constraint is imposed on the classifier function: any partition of the feature space is allowable. For any *k*-feature set {*x_i_*_1_, *x_i_*_2_,…, *x_ik_*}, the optimal classifier is the Bayes classifier relative to the feature-label distribution restricted to the subspace spanned by {*x_i_*_1_, *x_i_*_2_,…, *x_ik_*}. The best feature set, which in this case we write as $A_{k}^{\mathrm{bayes}}$ , consists of the *k* features whose corresponding Bayes error is minimal. Now suppose the classifier function is constrained, meaning the partition of each *k*-dimensional subspace is constrained. Then, for each feature set {*x_i_*_1_, *x_i_*_2_,…, *x_ik_*}, the optimal constrained classifier (optimal allowable partition) is the constrained classifier possessing the smallest error relative to the feature-label distribution restricted to the subspace spanned by {*x_i_*_1_, *x_i_*_2_,…, *x_ik_*} and $A_{k}^{\mathrm{best}}$ is a feature set for which this error is minimal. To illustrate, suppose the two *n*-dimensional class-conditional distributions are Gaussian with distinct means and covariance matrices. Then, for any *k*-dimensional subspace, the restricted distributions are also Gaussian and, to avoid degenerate cases, suppose they also possess distinct means and covariance matrices. If there is no classifier constraint, then the optimal classifier for any subspace is given by quadratic discriminant analysis (QDA) and $A_{k}^{\mathrm{bayes}}$ is the *k*-feature set whose QDA classifier has minimum error. Now, suppose we are constrained to linear classifiers. Then, for any *k*-dimensional subspace the optimal linear classifier is defined by the hyperplane possessing minimal classification error in that subspace and $A_{k}^{\mathrm{best}}$ is the *k*-feature set whose hyperplane classifier has minimum error.

For some feature-label distributions the Bayes classifier is known, so that $A_{k}^{\mathrm{bayes}}$ is known in the unconstrained case; however, optimal constrained classifiers are known to a much lesser extent. Consider feature selection for a constrained classifier where we do not know $A_{k}^{\mathrm{best}}$. One alternative is to use $A_{k}^{\mathrm{bayes}}$ in place of $A_{k}^{\mathrm{best}}$ .The plausibility of this approach rests on the assumption that we are using a constrained classifier to reduce design cost and that, given sufficient data, we would be taking an unconstrained approach in order to approximate the Bayes classifier. To wit, our real interest is in the best Bayes feature set and the overall process, feature selection and constrained function construction, is an attempt to approximate the Bayes classifier under practical experimental conditions.

Lacking an analytic solution for the best feature set, another way to proceed is to estimate it. Suppose we are considering a classification rule that produces classifiers in a function class *C* and, absent feature selection, Ψ is a consistent classification rule (either the one under study or a different one) relative to *C* for the feature-label distribution. Then, for any fixed feature set, *E*[Δ_*C,n*_] → 0 as *n* → ∞, so that according to Eq. 1, *E*[ε*_n_*] → ε_*A*_ + Δ_*C*_, the error of the optimal constrained classifier. This implies that, for a *k*-feature set *B_k_*_,*i*_ = {*x_i_*_1_, *x_i_*_2_,…, *x_ik_*}, the limiting error, as *n* → ∞, of the designed classifier for *B_k_*_,*i*_ closely approximates the error of the optimal classifier for *B_k_*_,*i*_. Thus, we can proceed in the following manner: (1) generate a large data set from the feature-label distribution and apply Ψ to each *k*-feature set *B_k_*_,*i*_ to design a classifier φ_*k*,*i*_; (2) compute the error of φ_*k*,*i*_, which approximates the error of the optimal classifier for *B_k_*_,*i*_; and (3) take the feature set corresponding to the minimum approximating error as an estimate, ${\hat{A}}_{k}^{\mathrm{best}}$ , of $A_{k}^{\mathrm{best}}$ . There are usually many close-to-optimal feature sets [19, 20]. From the viewpoint of feature selection, these are essentially equivalent. The main impediment to this approximation method is that, owing to computational reasons, it is limited to modest numbers of features, both potential and selected. Nonetheless, it is useful because if a feature selection does not perform well with *d* = 70, it cannot be expected to perform well with *d* = 1000.

The preceding approximation method can be used with real data sets, so long as the total sample size is sufficiently large. One approach is to infer a feature-label distribution from the sample data and then apply the preceding method using the inferred distribution, that is, by generating random samples from the inferred distribution. Note that unless some distributional model is used and we know a consistent classification rule for that model, one needs to employ a universally consistent rule. Another way is to view the data set as a large sample arising from some unknown feature-label distribution. We can then proceed as before with two modifications: (1) since the feature-label distribution is unknown, the classification rule must be universally consistent; and (2), whereas when the feature-label distribution is known we can either directly derive the errors of the designed classifiers or precisely estimate them by generating a second large independent data set, now we must use some training-data error-estimation procedure to estimate the classifier errors from the data set (which should provide decent results assuming a large data set). Whichever method is used, one faces the computational burden of obtaining ${\hat{A}}_{k}^{\mathrm{best}}$ .One approach is to develop a feature-selection test-bed by finding approximate best feature sets using high-performance computing [21]. In general, taking a non-model-based approach is weaker than a model-based approach since one does not actually know the exact conditions (feature-label distribution) under which performance has been verified and, perhaps more importantly, performance cannot be analyzed relative to model parameters, such as feature variance, which provides key knowledge for experimental design.

## PERFORMANCE COMPARISON WITH THE BEST FEATURE SET

When feature-selection is employed, it constitutes part of the overall classification rule, the other part being rule construction. If we fix the rule construction, say, linear support vector machine, linear discriminant analysis, or neural network, then we can focus on the performance of a particular feature-selection algorithm relative to the construction rule. Two basic related questions arise [22]: (a) Can one expect feature selection to yield a feature set whose error is close to that of an optimal feature set? (b) If a good feature set is not found, should it be concluded that good feature sets do not exist? The second question is confronted by researchers whenever they believe discrimination should be possible but are unable to find a good feature set. These two questions translate quantitatively into questions concerning conditional expectation. (a) Given the error of an optimal feature set, what is the conditionally expected error of the selected feature set? (b) Given the error of the selected feature set, what is the conditionally expected error of the optimal feature set? Rather than using the conditional expectation, one can take a simpler route and look at the linear regression in both cases. A global measure is given by the difference, $E\left[\varepsilon_{n,k}\right]-E\left[{\text{ε}}_{n,k}^{\mathrm{best}}\right]$ , between the expected errors of the selected and best feature sets. All of these performance measures depend on the joint distribution of the random vector $\left({\text{ε}}_{n,k},{\text{ε}}_{n,k}^{\mathrm{best}}\right)$ .

To illustrate performance comparison relative to the joint distribution, we employ a feature-label distribution consisting of two equally likely Gaussian class-conditional distributions. Letting **I** denote the identity matrix, class 0 has mean at (0, 0,…, 0) with ${Κ}_{0}=\sigma_{0}^{2}\text{I},{\text{σ}}_{0}=0.8$ , and class 1 has mean at (*a*_1_, *a*_2_,…, *a_d_*), where *a*_1_, *a*_2_,…, *a_d_* have been selected according to a beta distribution, Beta (0.75, *b*), where *b* is uniformly distributed over [1, 3], with ${\text{Κ}}_{1}={\text{σ}}_{1}^{2}\text{I},{\text{σ}}_{1}=1.2$.With these covariance matrices, the features are independent. In this model, the features can be ranked according to the values of *a*_1_, *a*_2_,…, *a_d_*, better features resulting from larger values. The optimal classifier for the model is determined by the QDA discriminant

(3) $$d_{j}\left(\text{x}\right)=-{\left(\text{x}-{\text{u}}_{\text{j}}\right)}^{\prime }{\text{K}}_{j}^{-1}\left(\text{x}-{\text{u}}_{\text{j}}\right)-\text{log}\left(\text{det}\left[{\text{K}}_{j}\right]\right)+2logf\left(j\right)$$

for *j* = 0, 1, where point **x** is classified as *Y* = 1 if *d*_1_(**x**) > *d*_0_(**x**). QDA is the Bayes classifier for the model. QDA is applied to sample data using the sample means and sample covariance matrices in place of the means and covariance matrices. Sequential floating forward search (SFFS) [23] is used to select the features, which will be compared with the best features from the model. The total number of features is *d* = 500. Feature sizes run from 2 to 30, and sample sizes run from 40 to 150 in increments of 10.

We focus on the regression of the true error, ε_*best*_, for the best features found from the model on the true error, ε_*SFFS*_, for the SFFS features (see the [22] for the examples of the reverse regression). Parts (a) and (b) of Fig. (**1**) show the regressions for *k* = 5 features and sample sizes *n* = 50 and *n* = 100, respectively, for the sample-designed classifier. The dots on the axes show the average errors. For *n* = 50 the correlation coefficient is 0.35, which is very low, and goes up to 0.56 for *n* = 100, still not very high. For the regression coefficient, there is little change in going from 0.21 to 0.27, which means that in both cases, a large SFFS-feature error cannot be used to indicate a similarly large best-feature error. Parts (c) and (d) of Fig. (**1**) correspond to parts (a) and (b), respectively, except for these the classifiers have been found from the feature sets using the feature-label distribution not the sample data. Note the tightening of the scatter plot, reflected by better correlation, but regression is still poor. Fig. (**2**) shows the correlation as a function of feature-set and sample sizes. Parts (a) and (b) show sample and distribution design, respectively. Note that there is correlation peaking with respect to feature-set size for sample design but no peaking for distribution design, which is analogous to error peaking for the two kinds of design.

## PEAKING PHENOMENON

For each *k*, the constraint cost in Eq. 2 is relative to the best feature set among all feature sets of size *k*. Since finding the best feature set from the sample data involves feature selection and is part of the classification rule, feature selection contributes to the design cost. Historically, the peaking phenomenon has typically been considered absent feature selection by choosing a canonical ordering of the features, *x*_1_, *x*_2_,…, *x_d_*, and letting *C*_*k*_ be the family of classifiers based on features *x*_1_, *x*_2_,…, *x_k_*. One way is to order the features according to their individual performances, say, from best to worst. In Gaussian models they might be ordered according to their place in the covariance matrix (not necessarily their row ordering). In this way, for a given *k*, there is no feature selection in the classification rule and therefore there is no design cost for feature selection. It should be kept in mind that, if the features are ordered from best to worst individual performers, this does not mean that {*x*_1_, *x*_2_,…, *x_k_*} is the best *k*-feature set. For instance, it may be that the best *k*-feature set does not include any of the best *k* individual performers. While the use of a canonical ordering ignores the practical problems of feature selection, it provides a framework in which peaking can be studied absent confounding by feature selection.

To illustrate peaking (finding the optimal number of features) when the features are ordered, we consider two models in which the class-conditional distributions are Gaussian and equally likely. For the *quadratic model*, the class-conditional distributions possess different covariance matrices, **K**_0_ and **K**_1_, and the Bayes classifier results from QDA. For the *linear model*, the classes possess identical covariance matrix **K**, and the Bayes classifier results from linear discriminant analysis (LDA), defined by Eq. 3 with **K**_*j*_ = **K**. The maximum dimension is *d* = 30, so that the peaking phenomenon can only arise in the graphs for which peaking occurs with less than 30 features. We assume the blocked covariance structure

(4) $$\text{K}=\sigma^{2}\left[\begin{array}{c} \begin{array}{ccccccccc} 1 & \rho & \rho & & & & & & \\ \rho & 1 & \rho & & & & & 0 & \\ \rho & \rho & 1 & & & & & & \\ & & & . & & & & & \\ & & & & . & & & & \\ & & & & & . & & & \\ & & & & & & 1 & \rho & \rho \\ & 0 & & & & & \rho & 1 & \rho \\ & & & & & & \rho & \rho & 1 \end{array} \end{array}\right]$$

of dimension 30. Features within the same block have correlation coefficient ρ and features in different blocks are uncorrelated. There are *m* groups, with *m* being a divisor of 30, so that *r* = 30/*m*. We denote a particular feature by *x_ij_*, where *i*, 1 ≤ *i* ≤ *m*, denotes the group to which the feature belongs and *j*, 1 ≤ *j* ≤ *r*, denotes its position in the group. We list the features in the order *x*_11_, *x*_21_,…, *x_m_*_1_, *x*_12_,…, *x_mr_*. In the quadratic model, 2**K**_0_ = **K**_1_ = **K**. The class means are **μ**_0_ = (0, 0,…, 0) and **μ**_1_ = (1, 1,…, 1). In all cases, the variance σ^2^ is set to give a Bayes error of 0.05 when the feature-set size is 10.

Fig. (**3**) (a) shows the results for LDA with the linear model, *m* = 5 groups, and ρ = 0.125 [16]. Note that the sample size must exceed the number of features to avoid degeneracy. Peaking occurs with very few features for sample sizes below 30, but exceeds 30 features for sample sizes above 90. In part (b), the features are highly correlated and, even with a sample size of 200, the optimal number of features is only 8. Similar results are observed for the nonlinear model. The concave behavior and increasing number of optimal features in parts (a) and (b) correspond to the usual understanding of peaking. Part (c) shows results for the 3-nearest-neighbor (3NN) classifier on the quadratic model with a single group and ρ = 0.25. The optimal-feature-number curve is not increasing as a function of sample size. The optimal feature size is larger at very small sample sizes, rapidly decreases, and then stabilizes as the sample size increases. To check this stabilization, the 3NN classifier has been tested on the quadratic model case in the figure for sample size up to 5000. The result shows that the optimal feature size increases very slowly with sample size. For *n* = 100 and *n* = 5000, the optimal sizes are 9 and 10, respectively. Finally, in part (d), with ρ = 0.25 in the quadratic model and using a linear support vector machine, not only does the optimal number of features not increase as a function of sample size, for fixed sample size the error curve is not concave. For some sizes the error decreases, increases, and then decreases again as the feature-set size grows, thereby forming a ridge across the error surface.

In practice, the features are not ordered and feature sets of increasing size are selected *via *a feature-selection algorithm. While we may then order the number of features, it is often the case that the feature set corresponding to *k* features is not a subset of the feature set corresponding to *k* + 1 features. What we observe in this case is that there is no clearly defined peaking phenomenon, the error curve taking various shapes with respect to the number of features [17]. The error curves in Fig. (**4**) illustrate the situation. Features in all three models are comprised of 60 useful features and 1140 noise features, all of which are randomly permuted. The noise features are modeled as independent random Gaussian variables with mean 0 and variance σ^2^. The useful features also follow a Gaussian distribution, but the exact set-up depends on the specific model. In model M1n, all features are uncorrelated and both classes have the same covariance structure; in M4n, all features are correlated the same way and the covariance matrices for the two classes differ by a constant factor; and in M5n, again the two covariance matrices are the same, but the features are grouped into 6 blocks such that within each block, all features are correlated in the same way and features from different blocks are uncorrelated. The sample size is 60. Fig. (**4**) shows three kinds of commonly observed curves [17], each being constructed using a feature-selection algorithm and a classifier rule: convex (LDA with t-test in model M1n), slow slope (3NN with ReliefF in model M5n), and plateau (3NN, with SFFS in model M4n).

## HIGH-DIMENSIONAL MODELS

High-throughput genomic and proteomic technologies require the discovery of discriminating features from among thousands of features, the vast majority of which contribute virtually no discriminatory power and act only as confounding variables that obscure good features. To illustrate the scenarios encountered in high-dimensional feature selection, we consider a model that emulates the situation in genomic data [24]. Fig. (**5**) provides a symbolic demonstration of how the model is constructed. The model describes an equal-probable two-class feature-label distribution of feature size 20,000. There are 20 global markers that follow a Gaussian distribution in each class: classes 0 and 1 are distributed as *N*(**0**^20^, **K**^global^) and *N*(**1**^20^, **K**^global^), respectively, where **0**^*n*^ and **1**^*n*^ represent an all-0 vector and an all-1 vector, both of length *n*, respectively, and **K**^global^ is the covariance matrix, which is identical in both classes. To emulate cases like the subtypes or stages of a certain disease, we further assume that there are two equal-probable subclasses in class 1. The two subclasses are mutually exclusive, so that each sample point in class 1 belongs to one and only one subclass. There are 80 heterogeneous markers, whose distributions are Gaussian: subclass 0 is distributed as *N*([**0**^40^, **1**^40^], **K**^hetero^), subclass 1 is distributed as *N*([**1**^40^, **0**^40^], **K**^hetero^), and class 0 is distributed as *N*(**0**^80^, **K**^hetero^), where **K**^hetero^ is the covariance matrix, which is identical in all distributions. For the structure of **K**^global^ and **K**^hetero^, we assume the blocked covariance structure defined in Eq. 4. There are *m* = 4 groups for global markers and *m* = 16 groups for heterogeneous markers of each subclass. We let σ = 0.7 and ρ = 0.8 for both **K**^global^ and **K**^hetero^. All remaining features are non-markers. We have 2000 independent high-variance non-markers that can be viewed as features regulated by mechanisms unrelated to the phenotype of interest. The distribution of each high-variance non-marker is a Gaussian mixture: *pN*(0, σ) + (1 - *p*)*N*(1, σ), with *p* randomly selected for that high-variance non-marker from a uniform distribution. Other non-markers are independent Gaussian variables that have zero mean and variance σ^2^. We let σ = 0.7 for all non-markers.

For comparison, we consider two filter methods: t-test, a univariate method, and ReliefF, a multivariate method, and two wrapper methods: SFS and SFFS. For the wrapper methods, we use a two-stage feature-selection scheme to avoid prohibitive simulation time. In the first stage, a filter method reduces the candidate feature size to 1000. In the second stage, a wrapper method is applied to search for the best feature set. The sizes of selected feature sets run from 1 to 30. LDA classifiers are constructed and tested accordingly. Fig. (**6**) shows the results at three different sample sizes. We see that the t-test has better performance when the sample size is small (*n* = 60), but with significant peaking. In comparison, SFS and SFFS cannot take advantage of the high correlation among features at a small sample size but catch up when the sample size is sufficiently large (*n* = 120 and 180). Although ReliefF has worse performance than the t-test, ReliefF-based SFS and SFFS show comparable performance to their t-test-based counterparts.

## PERFORMANCE ANALYSIS USING REAL DATA

We now demonstrate performance analysis using patient data, where we take the full data set to be an empirical distribution from which samples are taken and the best feature set is determined using a feature-selection test-bed [21]. The data are from a microarray-based classification study that analyzes microarrays prepared with RNA from breast tumor samples from 295 patients [25]. Using a previously established 70-gene prognosis profile [26], a prognosis signature based on gene expression is proposed in [25]. Of the 295 microarrays, 115 belong to the ‘good prognosis’ class and 180 belong to the ‘poor-prognosis’ class. We use the intensity gene-expression values associated with the 70 genes.

**Fig. (1):**
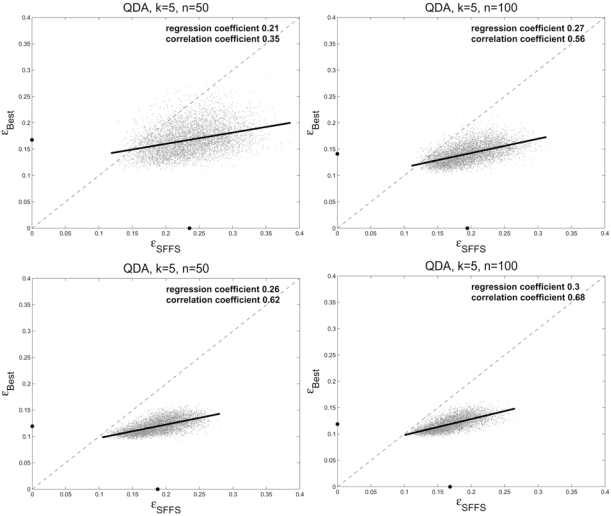
Scatter plots and regression lines for regressing the error of the best features on the error of the SFFS features, QDA classifier and 5 features: (**a**) *n* = 50, sample design; (**b**) *n* = 100, sample design; (**c**) *n* = 50, distribution design; (**d**) *n* = 100, distribution design.

We consider regression of the true error, ε_*best*_, for the best 5-gene feature set on the true error, ε_*SFFS*_, for the 5-gene feature set found by SFFS for LDA classification. A total of 200 sample sets of size *n* = 50 are randomly drawn with replacement from the data, with the remaining 245 samples being held-out each time for estimating the true errors. The scatter plot and regression line are shown in Fig. (**7**) (corresponding to Fig. **1**), where we see that the line is essentially horizontal, meaning no regression, and the correlation coefficient is 0.02.

## CONCLUSION

The behavior of feature-selection algorithms is very complicated and performance depends strongly on the classification rule, feature-label distribution, and sample size. One algorithm may outperform another for a particular distribution or sample size, but be significantly outperformed on a different distribution or even on the same distribution for a different sample size. Peaking, which has been recognized for forty years, is an extremely complex phenomenon, has no standard form, and cannot safely be generalized from ordered to non-ordered features. Perhaps most importantly, in small-sample settings, especially in the presence of high dimensionality, there is often little correlation between the errors for the selected and best feature sets. Owing to its importance in contemporary high-throughput biological datasets, there needs to a serious effort to understand feature selection.

Given the current lack of understanding, it is prudent to be conservative when performing feature selection with small samples and keep feature sets small. Not only will this help to avoid peaking, it will also make error estimation more accurate. In addition, rather than select a single feature set, one can report a collection of feature sets that appear to perform well, recognizing that many of them may actually not be good but that there is greater likelihood that there will be good ones among the collection. Such an approach takes advantage of the observations in [19, 20] that there are often many good small feature sets. If feature sets are kept small and the number of potential features is trimmed by prior knowledge, one can exhaustively evaluate all feature sets. This approach has been taken in several studies, where feature sets are kept to a maximum of three features and a list of the top feature sets based on the error estimates is reported [27-29].

If one wishes to use a larger number of features and there is no pre-existing literature supporting the efficacy of the experimental protocol being employed, then the overall classification rule, including the feature-selection algorithm, should be tested on a model that the experimenter believes is somewhat representative of the population for the data. The test should use the number of potential features, the feature set size, the sample size, and the error estimator for the experiment. The test can be performed for any or all of the performance criteria discussed in this paper. Without such a performance characterization, one lacks the epistemological ground on which to draw conclusions from the analysis, since the scientific meaning of the analysis depends on the mathematical properties of methods used in the analysis [30].

## Figures and Tables

**Fig. (2):**
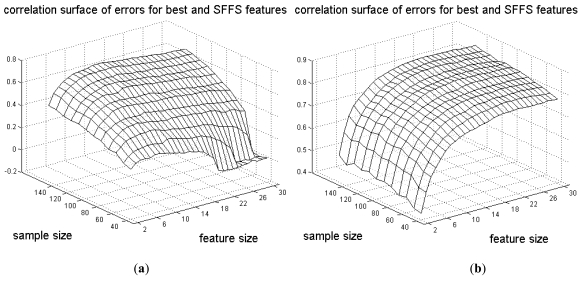
Correlation coefficient as a function of the sample size and number of features: (**a**) sample-based classifier design; (**b**) distributionbased classifier design.

**Fig. (3):**
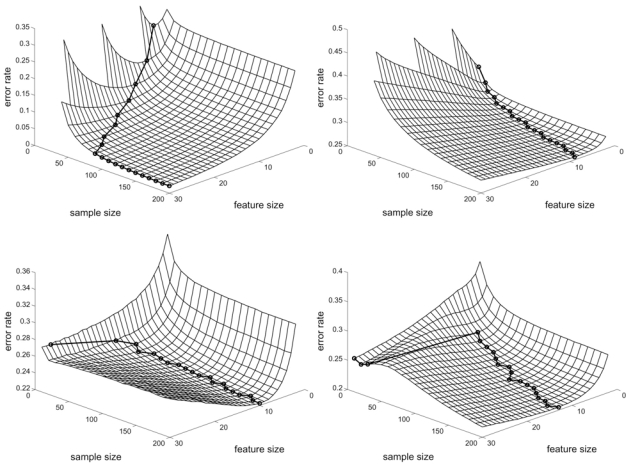
Optimal number of features: (**a**) for LDA in linear model with slightly correlated features; (**b**) for LDA in linear model with highly correlated features; (**c**) for 3NN in quadratic model; (**d**) for linear support vector machine in quadratic model.

**Fig. (4):**
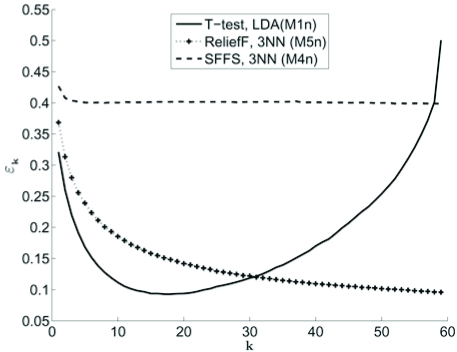
Three types of peaking in the presence of feature selection: convex (solid line), slow slope (“+” marked line), and plateau (dashed line).

**Fig. (5):**
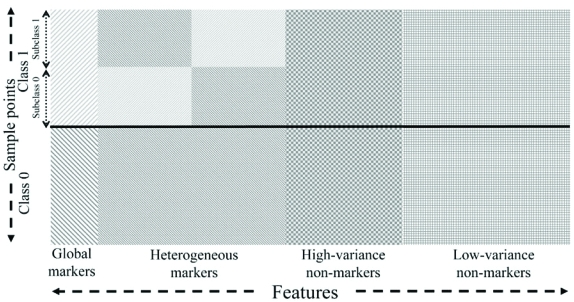
Symbolic demonstration of the high-dimension model.

**Fig. (6):**
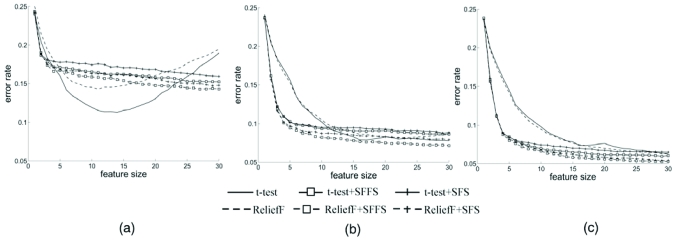
LDA error rates of different feature selection methods at different sample sizes: (**a**) 60; (**b**) 120; (**c**) 180.

**Fig. (7):**
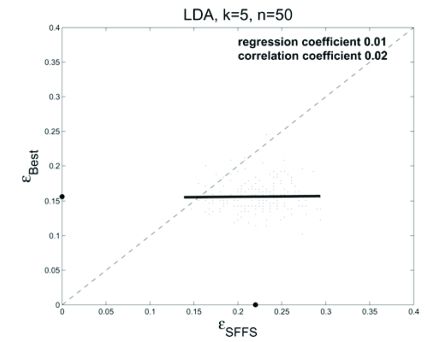
Scatter plot and regression lines for regressing the error of the best features on the error of the SFFS features for the LDA classifier with 5 features using real patient data.

**Table 1:** Feature Selection Studies: Sample Size Column Indicates the Total Sample Size. The Actual Number Used for Training Depends on the Criterion Function Used

Paper	Data Set Name	# of Class	# of Features	Sample Size	Criterion Function
Jain and Zongker 1997	Kittler’s synthetic data	2	20	2000	Mahalanobis
	SAR data	2	18	~11000	Split
Kudo and Sklansky 2000	SAR	3	10	285	LOO
	Vehicle	4	18	~800	1 x CV-9
	Mammogram (small)	2	19	86	LOO
	Kittler’s synthetic data	2	20	2000	Mahalanobis
	Mushroom (small)	2	29	1000	LOO
	Sonar (small)	2	40	208	LOO
	Sonar (large)	2	60	208	LOO
	Mammogram (large)	2	65	86	LOO
Kestler and Müssel 2006	Golub Data Set	2	3051	72	LOO 10 x CV-5 10 x CV-10
	Khan Data set	4	2308	63	
	Diagnostic Chip Data set	2	169	62	
Jeffery *et al*. 2006	DLBCL	2	7129	77	Resubstitution 10 x Hold-out 50% 10 x Hold-out 20 10 x Hold-out 10
	Prostate	2	12625	102	
	Colon	2	2000	62	
	Leukaemia (Golub Data Set)	2	7129	72	
	Myeloma	2	12625	173	
	ALL.1	2	12628	128	
	ALL.2	2	12628	125	
	ALL.3	2	12628	100	
	ALL.4	2	12628	93	

The criterion functions are: **Mahalanobis**: Mahalanobis distance; **Split**: data is split equally into training and testing sets; **LOO**: leave-one-out; **m x CV-n**: *n*-fold cross-validation repeated for *m* times; **m x Hold-out n**: hold-out *n* sample points and testing on the remaining, repeated for *m* times; **Resubstitution**: Resubstitution method.
